# Recent insights of T cell receptor-mediated signaling pathways for T cell activation and development

**DOI:** 10.1038/s12276-020-0435-8

**Published:** 2020-05-21

**Authors:** Jeong-Ryul Hwang, Yeongseon Byeon, Donghwan Kim, Sung-Gyoo Park

**Affiliations:** School of Life Sciences, Gwangju Institute of Science and Technology (GIST), Gwangju, 61005 Korea

**Keywords:** Signal transduction, Cellular immunity

## Abstract

T cell activation requires extracellular stimulatory signals that are mainly mediated by T cell receptor (TCR) complexes. The TCR recognizes antigens on major histocompatibility complex molecules with the cooperation of CD4 or CD8 coreceptors. After recognition, TCR-induced signaling cascades that propagate signals via various molecules and second messengers are induced. Consequently, many features of T cell-mediated immune responses are determined by these intracellular signaling cascades. Furthermore, differences in the magnitude of TCR signaling direct T cells toward distinct effector linages. Therefore, stringent regulation of T cell activation is crucial for T cell homeostasis and proper immune responses. Dysregulation of TCR signaling can result in anergy or autoimmunity. In this review, we summarize current knowledge on the pathways that govern how the TCR complex transmits signals into cells and the roles of effector molecules that are involved in these pathways.

## Immunology: The signals that turn on T cells

Researchers are unravelling the molecular signaling pathways that activate T cells, white blood cells which play a central role in the immune response. Sung-Gyoo Park and colleagues at the Gwangju Institute of Science and Technology in South Korea review the most recent insights and their significance for learning how to regulate the immune response in order to treat autoimmune disorders and other diseases. T cells are activated when a cluster of proteins on the cell surface, the T cell receptor complex, binds to molecules called antigens derived from pathogenic organisms or present on diseased cells. In autoimmune diseases, however, the receptor can be activated by the body’s own cells, initiating a damaging attack. Recent progress is identifying many specific molecules and their interactions in the cascades of signals that stimulate T cells following binding to antigens.

## Introduction

**Fig. 1:**
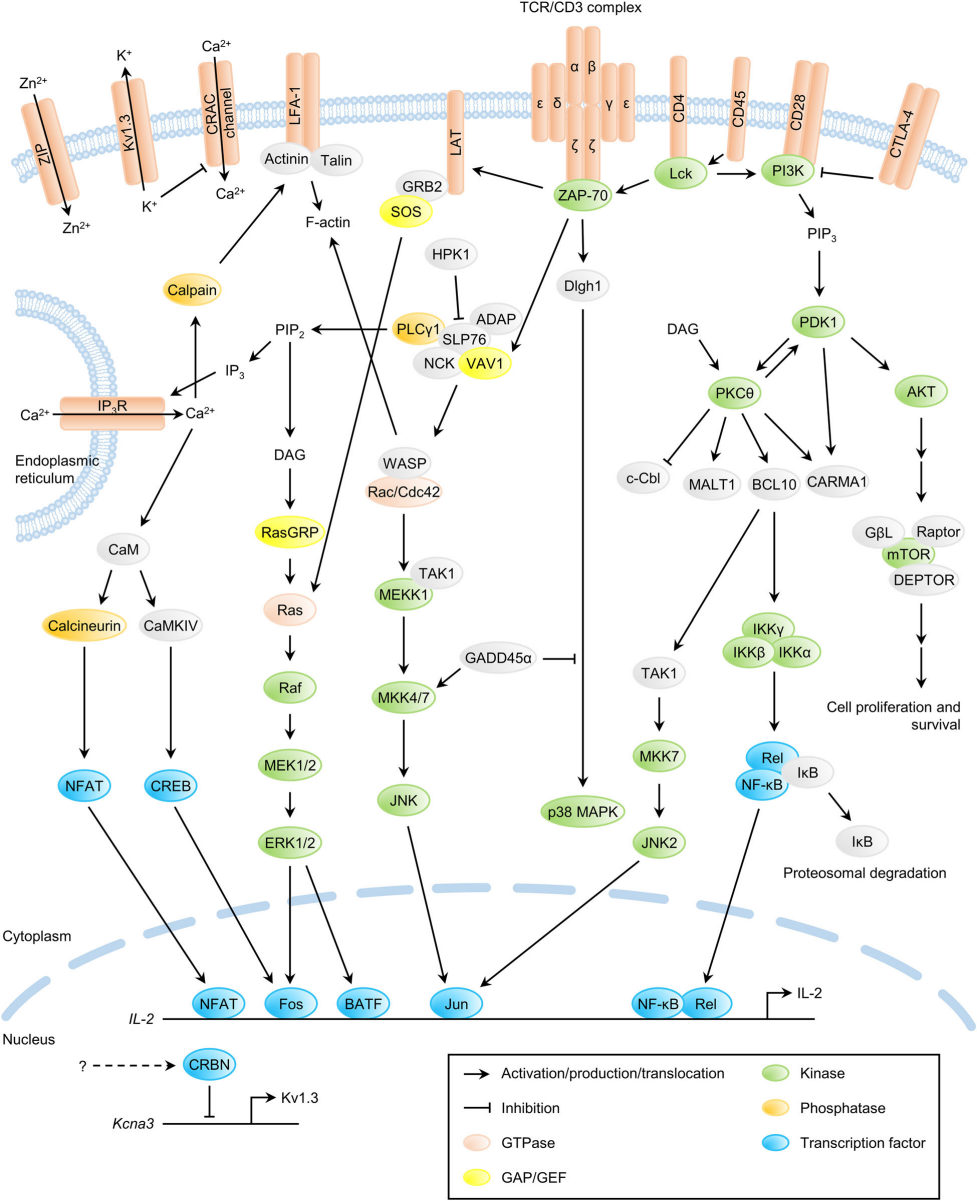
Overview of T cell receptor signaling cascades. ADAP adhesion and degranulation-promoting adapter protein, BCL B cell lymphoma, Cbl casitas B-lineage lymphoma, CaM calmodulin, CaMKIV calcium/calmodulin-dependent protein kinase type IV, CARMA1 caspase recruitment domain-containing membrane-associated guanylate kinase protein 1, Cdc42 cell division control protein 42, CRAC calcium release-activated calcium, CRBN cereblon, CREB cAMP response element-binding protein, CTLA-4 cytotoxic T lymphocyte-associated protein-4, DAG diacylglycerol, DEPTOR DEP domain-containing mTOR-interacting protein, Dlgh1 discs large homolog 1, ERK extracellular signal-related kinase, F-actin filamentous actin, GADD45α growth arrest and DNA damage inducible alpha, GβL G protein beta subunit-like, GRB2 growth factor receptor-bound protein 2, HPK1 hematopoietic progenitor kinase 1, IKK IκB kinase, IP_3_ inositol triphosphate, IP_3_R IP_3_ receptor, JNK c-Jun N-terminal kinase, LAT linker for activation of T cells, Lck lymphocyte-specific protein tyrosine kinase, LFA-1 lymphocyte function-associated antigen-1, MALT1 mucosa-associated lymphoid tissue lymphoma translocation protein 1, MAPK mitogen-activated protein kinase, MEK MAPK/ERK kinase, MEKK MAP kinase kinase kinase, MKK MAP kinase kinase, mTOR mammalian target of rapamycin, NCK noncatalytic region of tyrosine kinase adaptor protein 1, NF-κB nuclear factor-κB, NFAT nuclear factor of activated T cells, PDK1 phosphoinositide-dependent kinase 1, PI3K phosphoinositide 3-kinase, PIP_2_ phosphatidylinositol 4,5-bisphosphate, PKCθ protein kinase C theta, PLCγ1 phospholipase C gamma 1, Raptor regulatory-associated protein of mTOR, RasGRP Ras guanyl-releasing protein 1, SLP76 Src homology 2 domain containing leukocyte protein 76, SOS son of sevenless, TCR T cell receptor, WASP Wiskott–Aldrich syndrome protein, ZAP-70 zeta chain of TCR-associated protein 70, ZIP Zrt- and Irt-like protein.

As antigens enter the body, they are processed and presented by major histocompatibility complex (MHC) molecules expressed on the surface of antigen-presenting cells and recognized by T cell receptors (TCRs) on the surface of T cells. TCR signaling, in cooperation with signaling pathways induced by cytokines, costimulatory molecules, chemokines, integrins, and metabolites, drives the differentiation of activated T cells into specific T cell subtypes^1^. This results in the generation of various types of T cells with different specialized functions. Effector T cells fight against pathogens at initial exposure, and memory T cells provide defense against future infection. CD4^+^ T cells can differentiate into specialized effector subtypes, including T helper type 1 (Th1), Th2, Th17, follicular helper T, and regulatory T (Treg) cells. These subtypes regulate the immune response to address diverse types of pathogens. By generating specific T cell subtypes, the immune system can fine-tune itself and protect against inappropriate activation. It must achieve a delicate balance of sufficient activation to control infectious agents while preventing autoimmunity. Thus precise regulation of the T cell activation process is crucial for overall immune homeostasis^2^. Recent data suggest that TCR signaling is crucial for T cell differentiation and memory. How the fate of T cell differentiation is regulated has been widely investigated^3^. T cells are part of the adaptive immune system and fight against various infections and cancers. However, abnormal T cell function can cause autoimmune and inflammatory diseases. Naive T cells are initially activated through their TCRs by antigen/MHC complexes expressed by antigen-presenting cells. Subsequent signals, including environmental cues and signaling through CD28 or other costimulatory receptors, are required for T cell activation. Various signaling pathways, including the Ras-extracellular signal-related kinase (ERK)-activator protein (AP)-1 pathway, the inositol triphosphate (IP_3_)-Ca^2+^-nuclear factor of activated T cells (NFAT) pathway, the protein kinase C (PKC)θ-IĸB kinase (IKK)-nuclear factor (NF)-κB pathway, and the tuberous sclerosis complex (TSC)1/2-mammalian target of rapamycin (mTOR) pathway, are involved in TCR signaling (Fig. 1). Furthermore, several membrane proteins, such as lymphocyte function-associated antigen 1 and linker for activation of T cells, regulate T cell activation and function. For example, lymphocyte function-associated antigen 1 mediates TCR-induced T cell migration and activation by recruiting actinin and talin for the polymerization of filamentous actin^4,5^. Wiskott–Aldrich syndrome protein and cell division control protein 42 are also involved in actin filament polymerization, the activity of which can be regulated by a protein complex composed of VAV1, noncatalytic region of tyrosine kinase adaptor protein, and adhesion and degranulation-promoting adapter protein, which associates with linker for activation of T cells-Src homology (SH)2 domain-containing leukocyte protein 76^6^. However, hematopoietic progenitor kinase 1 inhibits this complex by phosphorylating SH2 domain-containing leukocyte protein 76^7^. Genetic/epigenetic controls also regulate T cell functions and activity^8–11^.

## Overview of TCR signaling and T cell development

### The TCR complex

The structural components of the TCR complex were revealed in the 1980s through intense investigation and advances in molecular biology and biochemistry techniques^12^. The TCR complex consists of TCRα/β chains and CD3γ/δ/ε/ζ subunits, which associate through hydrophobic interactions^13–15^. Somatic VDJ recombination enables the generation of distinct TCRα and TCRβ beta chains, and TCRα/β heterodimers are responsible for antigen recognition by binding to peptide–MHC complexes^16,17^. CD3 transmits the TCR-triggered signal through immunoreceptor tyrosine-based activation motifs (ITAMs) in its cytoplasmic tail, but it is not directly involved in antigen recognition^18,19^. ITAMs are tandem duplications of a tyrosine-containing sequence (YXXL/I), and the CD3γ/δ/ε chains each contain one ITAM, while the CD3ζ chain contains three^20,21^. As a consequence of TCR engagement, ITAM phosphorylation is induced by protein tyrosine kinases (PTKs), which allow other effector molecules to interact with the TCR complex^21^.

### Protein tyrosine kinases

The importance of tyrosine phosphorylation in TCR signaling was revealed by studies using PTK inhibitors^22,23^. TCR engagement triggers the activation and recruitment of PTKs, including Src family PTKs such as Lck and Fyn and the Syk family PTK zeta chain of TCR-associated protein 70 (ZAP-70)^24^. Evidence from Lck- or Fyn-null mice shows that Lck is crucial for T cell development, while Fyn is not essential for T cell development, as other Src family kinases can compensate for Fyn^25–27^. Lck is regulated by C-terminal Src kinase-mediated phosphorylation at its Y505 residue, which switches Lck to an inactive state^28^. The CD45 tyrosine phosphatase dephosphorylates inhibitory phosphorylation at Y505 and dephosphorylates positive regulatory autophosphorylation at Y394, although less efficiently^29–31^. This tight regulation of Lck activity protects against hyperactivation of T cells and autoimmunity, thus maintaining T cell homeostasis^29,31^. Activated Lck or Fyn phosphorylates the tyrosine residues of the ITAMs in the CD3 subunits. Tyrosine phosphorylation of CD3ζ provides the binding site for ZAP-70 via its SH2 domain, and then Lck or Fyn activates ZAP-70 by phosphorylation^24,32^. Therefore, recruitment of ZAP-70 to the activated TCR complex results in the formation of a signaling complex at the plasma membrane by recruiting other proteins through phosphorylation or activation^24^.

### T cell development

T cells develop from thymus-migrant hematopoietic lineage cells, particularly common lymphoid progenitors or lymphoid-primed multipotent progenitors derived from the bone marrow or the fetal liver^33,34^. Developing T cells in the thymus progress through four double-negative (DN1–4) stages, then a double-positive (DP) stage, and finally mature into single-positive (SP) naive T cells^35^. DN1–4 cells are distinguished by their expression of CD44 and CD25: DN1, CD44^+^CD25^−^; DN2, CD44^+^CD25^+^; DN3, CD44^−^CD25^+^; and DN4, CD44^−^CD25^−36^. At the DN3 stage, a pre-TCR complex that consists of a pre-TCRα chain and a mature β chain first appears. The mature β chain in this complex is a product of somatic DNA rearrangement by recombination activating gene 1/2^37–40^. T cells with a functional pre-TCR are positively selected by β selection, and they undergo massive proliferation and begin to rearrange the *Tcra* gene^35,41^. At the DP stage, both the CD4 and CD8 coreceptors are expressed (CD4^+^CD8^+^), and the αβ TCR is formed by replacing the pre-TCRα chain with the TCRα chain^42^. DP T cells encounter other checkpoints: DP T cells expressing αβ TCRs that recognize their MHC molecules through *Tcra* rearrangement are positively selected, and self-reactive T cells are deleted through negative selection^43,44^. In addition, DP T cells with dysfunctional TCRs that cannot receive or transduce TCR-mediated signals undergo apoptosis, while the selected cells further develop into CD4 or CD8 SP cells^45^.

### The strength of TCR signaling and T cell differentiation

TCR stimulation is a fundamental step in most T cell responses. When TCRs are stimulated, the quality or quantity of the resulting signaling is affected by various factors, such as the strength and length of stimulation. Interestingly, differences in the affinities of stimulatory agonists for the TCR are sufficient to cause differences in T cell physiology. When naive CD4^+^ T cells are subjected to strong TCR stimulation, Th1 cell differentiation is favored over Th2 cell differentiation, both in vitro and in vivo^46,47^. Conversely, weak TCR signals favor Th2 cell differentiation^46,47^. Whether differences in TCR signaling strength affect Th17 cell differentiation remains controversial^48,49^. Importantly, the strength of TCR signaling also regulates Treg cell differentiation. Although thymus-derived Treg cells are induced by a broad range of antigen affinities, high TCR signaling strength preferentially induces thymus-derived Treg cell differentiation^50,51^. In addition, for peripherally derived Treg cells, a low level of a strong agonism is important for their stable induction^52^. A longer TCR–pMHC dwell time, as well as a high-affinity TCR, is positively related to follicular helper T cell differentiation^53,54^. Furthermore, weak TCR stimulation suffices for the generation or enhancement of memory CD8^+^ T cell function, while a longer TCR–pMHC interaction, high levels of an antigen, or a high affinity antigen are associated with robust proliferation^1,55,56^.

## Regulatory mechanisms in TCR signaling

### Positive TCR signaling pathways

#### The Ras-ERK1/2-AP-1 pathway

Ras proteins make up a family of small GTPases expressed in animal cells that includes H-Ras, N-Ras, K-Ras4A, and K-Ras4B^57^. These isoforms have conserved effector binding domains but different carboxy-terminal regions, which enables them to selectively associate with various cell membranes, resulting in their intracellular compartmentalization^57^. Ras functions as a binary signal switch: as Ras is switched on, it transmits signals to other proteins, turning on genes involved in cell growth, differentiation, and survival^58^. If Ras is permanently activated by mutation, it can signal constitutively in the absence of activating signals, resulting in cell transformation^59^. All Ras isoforms are expressed in lymphocytes and are involved in TCR signaling and T cell development and function^60^.

The ERK1/2 pathway is a downstream signaling pathway of Ras, and it can be activated by persistent Ras signaling^61^. ERK1/2 is regulated by a feedback mechanism targeting ERK1/2 itself or its upstream activators. ERK1/2 inactivation is controlled by mitogen-activated protein (MAP) kinase phosphatases, which have dual specificity for Ser/Thr and Tyr residues. ERK1/2 signaling has an important role in controlling T cell development, differentiation, and TCR-induced signal strength^62,63^.

AP-1 is a basic leucine zipper transcription factor composed of homodimers or heterodimers of Jun, Fos, and activating transcription factor (ATF). AP-1 activity is regulated by extracellular signals that repress or activate AP-1 transcription^64,65^. For example, the basic leucine zipper ATF-like transcription factor, which belongs to the AP-1 family, can regulate osteoarthritic cartilage destruction by controlling anabolic and catabolic gene expression in chondrocytes^66^. Basic leucine zipper ATF-like transcription factor/Jun heterodimers can bind to AP-1-binding sites and regulate gene expression. The AP-1 family is also involved in Th17 differentiation^67,68^.

As upstream signals including TCR, Lck/Fyn, ZAP-70, and growth factor receptor-bound protein 2/son of sevenless are transmitted to Ras, GDP on Ras is exchanged for GTP by son of sevenless^69,70^. Ras is activated by GTP exchange, resulting in the sequential activation of the kinases Raf, MAP kinase/ERK kinase 1/2, and ERK1/2, resulting in the transcription of c-Fos and JunB. This results in the formation of the AP-1 complex, which induces interleukin (IL)-2 transcription^71,72^. The c-Jun transcription factor can be activated through the Rac/cell division control protein 42-MAP kinase kinase 4/7-c-Jun N-terminal kinase pathway and related proteins^73–75^. In addition, p38 MAP kinase can also regulate the activity of ATF^75,76^.

#### The IP_3_-Ca^2+^-NFAT pathway

IP_3_ is formed when phosphatidylinositol 4,5-bisphosphate is hydrolyzed by phospholipase C. IP_3_ functions as a second messenger. When IP_3_ binds to its receptor on the membrane of the endoplasmic reticulum, Ca^2+^ is released from the endoplasmic reticulum into the cytosol, resulting in the activation of various signaling pathways^77^. The calcium release-activated calcium channel controls the intracellular Ca^2+^ concentration in lymphocytes^78^. Ca^2+^ is a universal second messenger in T cells. T cell proliferation, differentiation, and effector functions are regulated by Ca^2+^^79^. There are two types of Ca^2+^ signaling pathways in T cells, long term and short term. NFAT is a transcription factor that is activated by Ca^2+^ influx in T cells and involved in long-term Ca^2+^ signaling^80^. It induces gene expression alone or in cooperation with other transcription factors^80^. When antigen/MHC complexes bind to TCRs, PTKs are activated, resulting in the phosphorylation and activation of phospholipase C-γ1. The membrane phospholipid phosphatidylinositol 4,5-bisphosphate is hydrolyzed, generating IP_3_ and diacylglycerol (DAG)^81^. Ca^2+^ efflux is sensed by stromal interaction molecules 1 and 2. Stromal interaction molecule proteins form clusters known as puncta, which trigger Ca^2+^ influx via calcium release-activated calcium channels^82,83^. Ca^2+^ influx activates the NFAT pathway via Ca^2+^-related proteins^84^. An increase in Ca^2+^ can also facilitate the formation of actin filaments^85^. A recent study demonstrated that deletion of *Crbn*, the gene encoding cereblon, in CD4^+^ T cells increases Kv1.3 channel expression and consequent Ca^2+^ flux, resulting in stronger NFAT activation after TCR stimulation^9^, implying that CRBN regulates TCR-induced NFAT activation. In addition, a previous study showed that zinc can regulate TCR signaling pathways and cytokine production by activated T cells^86^.

#### The PKCθ-IKK-NF-κB pathway

After T cell activation, the serine/threonine-specific PKCθ is recruited to the immunological synapse^87^. Then a signaling complex composed of caspase recruitment domain-containing membrane-associated guanylate kinase protein 1 (CARMA1), B cell lymphoma/leukemia 10 (BCL10), and mucosa-associated lymphoid tissue translocation protein 1 (MALT1) is formed in the cytoplasm^88^. PKCθ was the first PKC family member to be found to be recruited to the immunological synapse. It plays an integral role in activating a range of signaling cascades, leading to transcriptional regulation in T cells^88–95^.

Phosphoinositide-dependent kinase 1 (PDK1) is an enzyme that is involved in various signaling pathways. It plays a crucial role in T/B cell development and survival, intestinal homeostasis, and immune tolerance^96–99^. NF-κB is a well-known target of PDK1. PDK1 is required for TCR-mediated activation of NF-κB and PKCθ; selective deletion of PDK1 in T cells abrogates activation of NF-κB and PKCθ^98^. Recently, it was shown that PKCθ induces phosphorylation of PDK1^100^, resulting in T cell activation and TCR-induced NF-κB activation^96–98,100–102^. PKCθ is involved in the activation of NF-κB^103^. When T cells are in the resting state, NF-κB exists in the cytoplasm bound to IκB. When TCR/CD28 ligation occurs, NF-κB signaling is activated. PDK1 efficiently activates PKCθ via the phosphoinositide 3-kinase pathway^104^. Activated PKC phosphorylates a serine residue located in the membrane-associated guanylate kinase domain of CARMA1^105^. Then BCL10 and MALT1 are recruited, resulting in the formation of the active CARMA1-BCL10-MALT1 signaling complex^106^. This promotes IKK complex activation and IκB degradation, which allows NF-κB to translocate to the nucleus, initiating the transcription of genes that are required for T cell activation^11,103,107–110^.

CARMA1, also called CARD11, is a scaffold protein that is considered a hallmark of IKK/NF-κB activation. CARMA1 contains several domains, including a caspase recruitment domain and coiled-coil, SH3, guanylate kinase, and PDZ domains^106^. Except for the PDZ domain, each of these domains is required for CARMA1 to activate NF-κB. CARMA1 is constitutively associated with the plasma membrane and recruited into lipid rafts after TCR stimulation^111^. CARMA1 activation is mediated by several mechanisms^106^, including phosphorylation. PKC phosphorylates CARMA1 between its coiled coil and PDZ domains after it is activated by TCR/CD28 ligation. Phosphorylated CARMA1 undergoes a conformational change, enabling it to associate with BCL10 and MALT1^3^.

#### TSC1/2-mTOR signaling

TSC1 and TSC2 are tumor suppressors. They heterodimerize and regulate downstream signaling^112^. mTOR is involved in T cell activation, differentiation, and function^113^. Rapamycin is an immunosuppressant that promotes G1 arrest and inhibits downregulation of the cyclin-dependent kinase inhibitor p27^113^. Treatment of T cells with rapamycin inhibits their proliferation and leads to anergy. The ability of rapamycin to promote Treg cell generation underlies its ability to induce T cell anergy^114^. mTOR is activated by various signals, including growth factors, nutrients, and cellular stress signals, and regulates the growth, proliferation, and survival of cells^113,115^. Two different mTOR complexes, mTORC1 and mTORC2, are involved in mTOR signaling^113^. mTORC1 and mTORC2 both include the scaffolding proteins Raptor and Rictor. Activation of mTORC1 results in phosphorylation of S6 kinase 1 and translation of 4E-BP1, while activation of mTORC2 results in phosphorylation of the kinase AKT^116^. Recently, a relationship between TSC1/2 and mTOR was reported. When TSC2 is phosphorylated by AKT, the GAP activity of the TSC1/2 complex is inhibited, resulting in the activation of the small GTPase Rheb and mTORC1 activation^113^.

### Inhibitory TCR signaling pathways

#### Phosphatases

Phosphorylation and dephosphorylation of TCR signaling molecules affect signaling complex formation and propagation of TCR signals. Similar to CD45 phosphatase, SH2 domain-containing protein tyrosine phosphatase (SHP-1) dephosphorylates Lck at Y394, resulting in its inactivation^117^. TCR signaling-mediated Lck activation results in SHP-1 activation via activated Lck phosphorylation of Y564 in SHP-1, which in turn dephosphorylates and inactivates Lck^118^, resulting in the attenuation of both early and late TCR signaling events. TCR signaling induces the expression of an adhesion molecule, carcinoembryonic antigen-related cell adhesion molecule 1, at a later time point^119^. The phosphorylation of an immunoreceptor tyrosine-based inhibitory motif in carcinoembryonic antigen-related cell adhesion molecule 1 recruits SHP-1, which dephosphorylates Lck, resulting in the termination of TCR signaling^120^. By contrast, under strong TCR–ligand binding conditions, ERK1/2 phosphorylate Lck at S59, preventing the recruitment of SHP-1 and sustaining TCR signaling for gene expression^121^. Suppressors of TCR signaling-1 and -2, which are TCR signaling-related phosphatases, dephosphorylate Syk and ZAP-70, respectively^122,123^. Mice lacking both suppressors of TCR signaling-1 and -2 show T cell hyperproliferation, enhanced activation of TCR signaling, and increased susceptibility to autoimmunity^123^. Furthermore, PTEN dephosphorylates PIP_3_, and dual specificity phosphatases dephosphorylate ERK2^124,125^. These observations suggest that dephosphorylation events in TCR signaling are important for the termination or negative regulation of TCR signaling cascades.

#### Ubiquitination and degradation

There is increasing interest in understanding the role of proteolytic mechanisms in the regulation of TCR signal transduction. Proteolysis is primarily caused by proteasomal or lysosomal processes. Many short-lived proteins selectively undergo ubiquitination before their proteasomal degradation. Ubiquitination results from the conjugation of ubiquitin to proteins through a series of enzymatic reactions^126^. Ubiquitination is initiated when the E1 ubiquitin-activating enzyme releases ubiquitin from the inactive state. Active ubiquitin is then transferred to an E2 ubiquitin-conjugating enzyme. Finally, the E3 ubiquitin ligase transfers activated ubiquitin from the E2 enzyme to the target protein. Thus the E3 ligase facilitates the actual attachment of ubiquitin to the substrate and therefore controls the specificity of substrate targeting^127^. Although many types of E3 ligases have been reported, the mechanisms determining their substrate specificity are not clearly understood^128^.

Ubiquitination is an important process in the regulation of T cell development, activation, and immune tolerance. Therefore, failure to regulate ubiquitination appropriately can lead to autoimmune and inflammatory diseases^129^. Several E3 ligases, such as casitas B-lineage lymphoma (Cbl), Itch, Deltex, and gene related to anergy in lymphocytes, are known to be involved in the regulation of TCR signaling via ubiquitination-mediated degradation of TCR signaling molecules, including CD3ζ, PKCθ, ZAP-70, phospholipase C-γ1, and phosphoinositide 3-kinase^130–133^. Itch ubiquitinates Jun, thereby reducing AP-1 activity^134^, while Cbl ubiquitinates CD3ζ via an adapter molecule, ZAP-70^135^. In contrast, Cbl-b and Itch inhibit the association between ZAP-70 and CD3ζ by conjugating K33-linked ubiquitin chains to CD3ζ, which does not lead to its degradation^136^. T cell anergy is also regulated by E3 ligases, including Cbl-b, c-Cbl, gene related to anergy in lymphocytes, and Itch^137^. The expression of gene related to anergy in lymphocytes is induced under anergic T cell conditions with decreased IL-2 production^71^. Roquin1/2 E3 ligases maintain immune tolerance by regulating T cell activation and differentiation appropriately^138,139^. Furthermore, ubiquitination plays a role in TCR-induced T cell activation. IκB ubiquitination and subsequent degradation of NF-κB are well-known processes^140,141^. However, before these can occur, NF-κB essential modulator (also known as IKKγ) must be ubiquitinated by the TRAF6 E3 ubiquitin ligase (K63-linked polyubiquitination), which contributes to the activation of the IKK complex^142–144^.

#### DAG kinases

DAG is an important signaling molecule involved in several signaling cascades. DAG kinases (DGKs) are lipid kinases that convert DAG to phosphatidic acid by phosphorylation, thereby regulating the subcellular DAG level^145^. Ten isoforms of mammalian DGKs have been identified, among which DGKα and DGKζ act as crucial regulators downstream of the TCR^146,147^. When DGKζ expression increases in T cells, TCR-induced Ras-ERK signaling is reduced^148^. In addition, T cells exhibiting loss of DGKα and/or DGKζ show increased TCR-induced signaling, including Ras-MAP kinase/ERK kinase-ERK-AP-1, PKCθ-NF-κB, and mTOR pathway signaling, leading to hyperactivation, impaired induction of anergy, and reduced antiviral responses in CD8^+^ T cells^149–154^. DGKα/DGKζ double-knockout mice show impaired T cell development, and phosphatidic acid treatment partially rescues T cell development, suggesting that DGKs not only terminate DAG signaling but also initiate phosphatidic acid-mediated signaling^155^.

## T cell development pathways

### Transcriptional control of T cell development

#### Ikaros

**Fig. 2:**
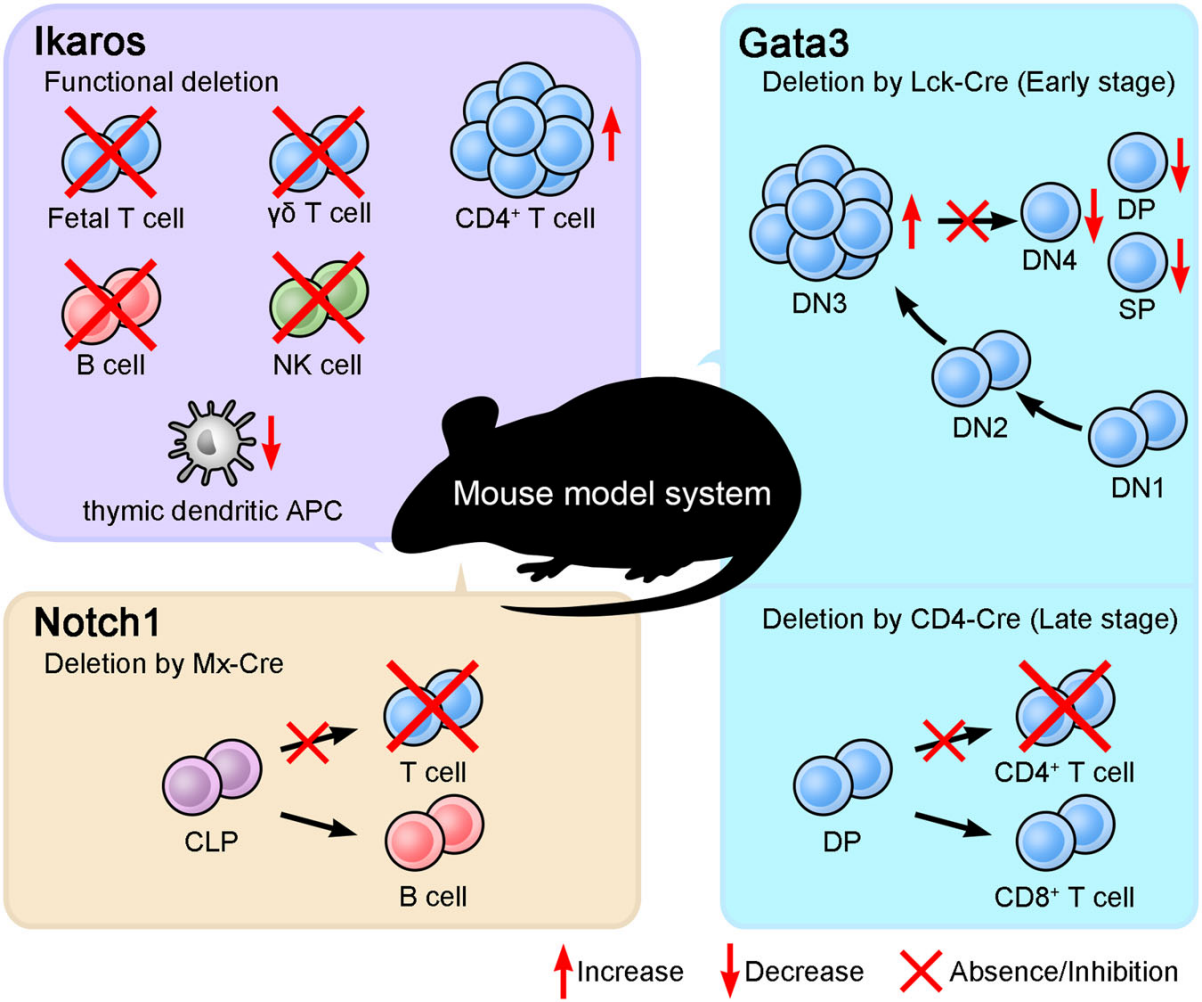
Transcriptional controls of T cell development. CLP common lymphoid progenitor, DN double negative, DP double positive.

Members of the Ikaros transcription factor family, including Helios and Aiolos, possess zinc-finger motifs^156–158^. They are most abundant in hematopoietic lineages and are mainly lymphocyte restricted^156,159^. Mice with a homozygous mutation in the DNA-binding domain of Ikaros lack T, B, and natural killer cells, as well as their earliest progenitors^160^. In another study, functionally Ikaros-null mutant mice were generated by deleting the C-terminal region to avoid a dominant-negative effect due to the loss of the DNA-binding domain^161^. These mice show an absence or large reduction in fetal T and B cells and in adult γδ T, B, natural killer, and thymic antigen-presenting cells and show aberrant proliferation and differentiation into CD4 lineage T cells postnatally^161^. These studies suggest that Ikaros promotes the differentiation of hematopoietic stem cells into lymphocytes and establishes early branch points in postnatal T cell development^160,161^ (Fig. 2). In addition, Ikaros regulates checkpoints in T cell development, such as β selection and the transition from the DP to the SP stage^162^.

#### GATA-3

GATA transcription factor family proteins contain zinc-finger motifs that recognize the consensus DNA sequence WGATAR (W represents A or T, and R represents A or G)^163,164^. GATA-3 was first identified as a regulator of the *Tcra* gene that binds to a Tα3 element in the *Tcra* enhancer^165^, but it also has a well-known role in Th2 cell differentiation^166,167^. In lymphoid lineage cells, GATA-3 expression is restricted to early hematopoietic precursors, immature/mature T cells, and natural killer cells^165,168,169^. One group assessed the role of GATA-3 in immune cell development using a *Rag2*^−/−^ blastocyst complementation system because homozygous deletion of *Gata3* is embryonic lethal and showed that GATA-3 is crucial for T cell development^170^. Another study using mice with a conditional deletion in Gata3 in early- or late-stage thymocytes showed arrest of the DN3 population with decreased DN4, DP, and SP populations or impaired differentiation into CD4^+^ T cells, respectively.^171^. These results imply that GATA-3 affects β selection and commitment to the CD4 SP lineage^171^ (Fig. 2). In the choice between the CD4 or CD8 lineage, GATA-3 can directly induce expression of the *Zbtb7b* gene, which encodes the transcription factor ThPOK^172^. ThPOK independently inhibits the differentiation of DP thymocytes into CD8 SP cells and promotes differentiation into CD4 SP cells in a GATA-3-dependent manner, suggesting that GATA-3 is an upstream regulator of ThPOK^172^. On the other hand, the exact role of GATA-3 in β selection is not completely known.

#### Notch

Notch was first identified as a regulator of cell fate decisions during neuronal and epidermal cell differentiation in Drosophila^173^. In mammals, it is a transmembrane receptor of the Delta/Serrate/Lag-2 family that interacts with membrane-associated ligands, Jagged 1/Serrate 1, Jagged 2/Serrate 2, Delta 1, Delta 2, and Delta 3^174^. Interaction between cells expressing Notch and cells expressing Delta/Serrate/Lag-2 ligands causes proteolytic cleavage of Notch, which migrates to the nucleus and releases intracellular domains that interact with recombination signal-binding protein for immunoglobulin kappa J region, leading to gene regulation^175,176^. Targets of activated recombination signal-binding protein for immunoglobulin kappa J region are incompletely characterized. One known target is hairy and enhancer of split 1, which is upregulated by Notch and acts as a transcriptional repressor.

Further evidence for the role of Notch in T cell lineage determination comes from experiments in which mice were reconstituted with bone marrow stem cells expressing constitutively active Notch1. The differentiation of stem cells into B cells was completely blocked in these mice, which developed a thymus-dependent population of T cells in the bone marrow^177^. In contrast, deletion of *Notch1* or inhibition of Notch1 signaling in hematopoietic stem cells drives B cell development, while T cell development is blocked^178–180^. Thus lymphocyte progenitor cells develop into T cells via Notch signaling, but without these signals, the B cell fate is chosen (Fig. 2).

## Conclusions

The signals initiated by the activated TCR complex play essential roles in T cell-mediated immune responses. In recent decades, extensive efforts by researchers and advances in molecular, genetic, and biochemical techniques have made it possible to elucidate the structure and signaling molecules of the TCR complex. Engagement of the TCR complex is a prerequisite for the initiation of the TCR signaling cascades that were summarized in this review. TCR signaling is important for many aspects of T cell regulation, including development, differentiation, activation, proliferation, and survival. Therefore, TCR signaling must be tightly regulated. In this regard, therapeutics have been developed that target the TCR complex, mainly for immune suppression. For example, muromonab-CD3 (orthoclone OKT3) is the first mouse anti-human CD3 monoclonal antibody to be used in the clinic. It binds to CD3ε in circulating T cells and elicits immune suppression by inducing apoptosis. In an attempt to reduce its side effects related to its mouse origin, chimeric or humanized anti-CD3 monoclonal antibodies, such as otelixizumab, teplizumab, and visilizumab, have been developed and are under clinical trials for the treatment of various diseases.

The regulation of TCR signaling is a complicated process and is controlled by a large number of effector molecules, and there are still many aspects of T cell activation and development that are poorly understood. The integration of TCR-induced signaling and CD28-induced signaling is relatively well understood, but the effect of imbalances between these two signaling cascades on T cell differentiation and function is not well understood. For example, strong CD28 signaling blocks Th17 differentiation. Thus there are unknown regulatory mechanisms controlling T cell-mediated immune responses. A more comprehensive understanding of these processes will enable us to therapeutically modulate immune responses for the treatment of autoimmune disease and other immune-related diseases.
